# Performance on an attention test is positively related to reading but negatively related to watching TV and playing video games in children

**DOI:** 10.1186/s12887-025-06260-w

**Published:** 2025-10-28

**Authors:** Tanja Poulain, Ricarda Schmidt, Wieland Kiess, Sarah Krause, Simone Golz, Christof Meigen

**Affiliations:** 1 LIFE Leipzig Research Center for Civilization Diseases, Leipzig University, Philipp- Rosenthal-Strasse 27, 04103 Leipzig, Germany; 2 Department of Women and Child Health, Hospital for Children and Adolescents, Center for Paediatric Research (CPL), Leipzig University, Leipzig, Germany; 3 German Center for Child and Adolescent Health (DZKJ), partner site Leipzig/Dresden, Leipzig, Germany; 4 Medical Center, Integrated Research and Treatment Center AdiposityDiseases, Behavioral Medicine Research Unit, Department of Psychosomatic Medicine and Psychotherapy, Leipzig University, Leipzig, Germany

**Keywords:** Attention, Continuous performance, Children, Media use, Reading

## Abstract

**Background:**

When exploring associations between attention skills and children’s media use, most previous studies relied on parental or self-reports of attention performance. In the present study, attention was assessed using a standardized computer test.

**Methods:**

The study was carried out as part of the LIFE Child cohort study conducted in Leipzig, Germany. A total of 1057 children from a younger (3-6.5 years) and an older age group (6.5–11 years) underwent a Continuous Performance Test (CPT). Parents reported on the children’s use of electronic media (watching movies/TV shows, playing video games) and reading frequency (listening to parents’ reading in the younger age group and autonomous reading in the older age group). Associations between CPT outcomes (omission and commission errors, reaction time variability) and use of different media and reading were assessed by applying linear regression analyses. Child age, sex and family income were included as covariates.

**Results:**

Watching movies/TV shows was significantly associated with a higher rate of errors of commission in the younger age group and with a higher rate of errors of omission in the older age group. Playing video games was associated with a higher rate of errors of omission in the older age group only. Reading, in contrast, was associated with a lower total error rate in the older age group.

**Conclusions:**

The results strengthen the assumption that the frequent use of electronic media is associated with poorer attention skills in children, while frequent reading is associated with better attention performance.

**Trial registration:**

Clinical trial number NCT02550236 (clinicaltrials.gov, date of registration: 2014-12-15).

## Background

Attention is the ability to focus on specific information, to sustain this focus, and to ignore irrelevant information [1]. It enables us to acquire and store information and, therefore, is essential for successful learning. Several behavioral factors are associated with children’s attention, e.g., sleep, diet, physical activity, and media use [2]. The present study investigates associations between attention performance and different types of media use in children, namely the use of electronic media and reading.

In previous studies investigating this relationship, attention performance was often assessed by asking parents (or teachers) to report on children’s skills. These reports might be prone to several biases, e.g., recall bias, reporting bias, or social desirability [3]. Also, parents, as well as children themselves, may lack a clear benchmark for evaluating performance. Another way of assessing attention is testing it using standardized tests. This approach is more objective and less biased, and therefore likely to provide a more accurate estimate of children’s performance. In the present study, attention performance was assessed by a Continuous Performance Test (CPT). A CPT is a standardized test in which participants sit in front of a screen and have to react (usually by pressing a key) to specific stimuli presented on the screen as quickly as possible, while ignoring all other stimuli. The first CPT was designed in 1956 to assess attentional control in adults with brain damage [4]. To date, the CPT is the most widely used attention test in research and practice [5]. Performance on the CPT has been shown to differ significantly between children with and without Attention Deficit Hyperactivity Disorder (ADHD), with children diagnosed with ADHD performing significantly worse [6, 7]. In addition, different CPT outcomes indicate different deficits, namely difficulties in impulse control (errors of commission), sustained attention (errors of omission), or attention stability (response time variability).

### The use of media and associations with attention

Children’s use of electronic media has increased in recent decades, with mobile and internet-enabled media devices becoming more and more popular, even in preschool-aged children [8, 9]. In the United States, children aged 0 to 8 years spend on average 2.5 h a day using screen-based media [9]. In Germany, the use of screen-based media is less frequent; 2- to 5-year-old children spend on average 1.5 h a day using different electronic media [10]. In 6- to 13-year-olds, however, screen time exceeds 2 h a day [11]. These averages are above the maximum daily screen time of 0.5–1 h recommended for preschoolers and children at primary school [12, 13] and the maximum of 2 h recommended for older children [12, 14].

The importance of limiting children’s screen time is underscored by studies linking the early usage of electronic media, especially watching TV, to parent-reported externalizing behavioral difficulties such as symptoms of hyperactivity and attention deficits [8, 15–19]. Some studies also showed associations between the use of electronic media and the performance in attention tests [20, 21]. According to a recent study, media use among children diagnosed with ADHD is also significantly higher than among healthy children [22]. Another recent study in 8- to 12-year-old children showed a negative association between screen exposure and functional connectivity between neural networks associated with basic attention skills [23], suggesting that the use of electronic media may be associated with neural changes.

Possible reasons for the association between the use of electronic media and attention-related behavioral problems in childhood are highlighted in a longitudinal study [24]: According to the excitement hypothesis, electronic media are very exciting, which might increase the desire for high stimulation and decrease the ability to engage in less exciting activities, e.g., tasks requiring sustained attention. The displacement hypothesis states that time spent on electronic media might displace time that could be spent on activities requiring and evoking more attentional control. Finally, the attraction hypothesis assumes that media devices are particularly appealing to children with externalizing behavior and/or attention problems because their need for stimulation is quickly satisfied. Findings of the longitudinal study suggest that attention skills and use of electronic media are mutually reinforcing [24].

Another way of media use is reading. In contrast to electronic media use, reading is a less popular media activity. In Germany, 15% of 6- to 13-year-old children read on a daily basis [11]. Given that reading (or listening to someone’s reading) is considered one of the most educationally valuable media activities [25, 26], this percentage is very low. While the use of electronic media has been related to more (externalizing) behavioral difficulties and poorer attention skills, reading skills, assessed per parent- or teacher reports or reading tests, have been shown to be associated with fewer teacher-reported externalizing and internalizing behavioral difficulties [26] and better parent- or teacher-reported attention skills [21, 27–31]. Findings of longitudinal studies suggest that attention skills in primary school-aged children, evaluated by children’s teachers, predict later reading competencies [28, 29]. At the same time, an intervention study showed that a 3-year reading intervention in middle school children improved not only reading but also attention skills, indicating that reading might also have an impact on attention [30]. There exist different theories for the link between reading and attention skills in children. According to the inattention as cause hypothesis, poor attention hinders early word reading, which in turn causes reading disabilities [29]. The phenocopy hypothesis states that symptoms of inattention are secondary to reading difficulties [32]. Finally, the correlated liabilities hypothesis states that both inattention and reading problems share the same risk factors, an assumption that is supported by genetic overlap between reading disorders and ADHD [33].

Importantly, previous studies on the association between reading and attention focused on reading skills instead of reading frequency or duration. In the present study, we assessed the time children spend reading or being read to, which reflects both the motivation to read and the duration of focused activity.

Based on previous study findings, we expected to observe significant positive associations between the time children spend using electronic media and the errors made in a CPT, but significant negative associations between the time spent reading and errors in the CPT.

## Methods

### Participants

This study was conducted at the Leipzig Research Center for Civilization Diseases (LIFE Child) at the University of Leipzig, Germany. The LIFE Child study is a child cohort study exploring child development from the prenatal period to the beginning of adulthood, with a specific focus on the development of non-communicable diseases such as obesity, allergies, and depression [34, 35]. Child participants have been recruited since 2011 through advertisements at different institutions, e.g., childcare centers and schools. While recruitment ends when children are 16 years old, annual follow-ups may take place until age 20. Inclusion criteria for participation in the LIFE Child study are personal interest and the absence of chronic, chromosomal, or syndrome diseases.

The data used in the present project were collected between 2021 and 2024 (as the media use questionnaire was only implemented in 2021). This time period included the Covid-19 pandemic. During that time (2020–2022), schools were temporarily closed to prevent infection, which has greatly changed children’s everyday lives, health, and media behavior. However, the data collected here was only gathered during periods when schools were open.

All children who participated in the CPT and whose parents provided information on their child’s media use and their family income were eligible for the analysis (*n* = 1191). Of these children, 746 had participated more than once. In these cases, only the first visit was considered. Children with invalid/cancelled CPTs (e.g., because they were unable to remain seated, *n* = 111) or missings in relevant parameters (*n* = 23) were excluded from further analysis, resulting in a final sample of 1057 3- to 11-year-old children (544 boys, 513 girls). In older children, parental reports on children’s media use were not available. The children were grouped into two age groups based on their age and the CPT version they completed: The younger age group comprised 440 3- to 6.5-year-old children (mean age = 5.0 years), who completed a simple CPT. The older age group consisted of 617 6.5- to 11-year-old children (mean age = 9.1 years), who completed a 1-back CPT, as described in more detail below.

The study protocol was designed in accordance with the Declaration of Helsinki and was approved by the Ethics Committee of the Medical Faculty of the University of Leipzig (Reg. No. 477/19-ek). All parents provided informed written consent before their child was included in the LIFE Child study.

### Measures

#### Outcome measure: CPT

All participants completed a CPT. The test was completed in a quiet room during the data collection day at the LIFE Child study center (after breakfast, before 12 pm). Only the child and a research assistant were present in the study room. The child sat in front of a computer screen, on which nine different stimuli were presented in random order. Depending on the child’s age, two different versions of the CPT were presented; the simple version (younger age group) or the 1-back version (older age group). The simple CPT was originally designed by Valiente et al. [36]. In this version, a total of 220 stimuli were presented (10 per block). Each of the 22 blocks included two targets (fish). The child was instructed to detect the fish as fast as possible by pressing the space bar. The ISI was 2000 ms. The 1-back test version used the same stimuli. However, to make the test more appropriate for older children, the task was more difficult. Children had to press the space bar each time the target stimulus (fish) appeared after another specific stimulus (a plane). The 1-back CPT existed in a slow version (ISI 2000 ms, 21 blocks of 10 stimuli) for children aged 6.5 to 9.5, and in a fast version (ISI 1333 ms, 28 blocks of 10 stimuli) for children aged 9.5 or older. There were alternating blocks with either one or two targets, resulting in 31 targets in the slow and 42 targets in the fast version. After 1500 ms (or 1000 ms in the fast version of the 1-back CPT), the stimulus was hidden until the next picture was presented to maintain a clear connection between the picture shown and the key pressed. The outcome variables were the rate of errors of commission, the rate of errors of omission, the total error rate, and the reaction time variability. Errors of commission occur when children react although the target is not shown and are an indicator of difficulties in impulse control. Errors of omission occur when children do not react although a target is shown and indicate difficulties in sustained attention. To obtain the error rate, the number of errors (commission or omission) was divided by the number of targets (for errors of omission), non-targets (for errors of commission), or all stimuli (for total error rate) presented in the test, multiplied by 100. Reaction time variability was reflected by the standard deviation (sd) of the reaction time in milliseconds (for correct clicks only) and indicates difficulties in attention stability.

#### Exposure variable: media use

A child’s media use was assessed in a digital questionnaire completed by a parent (usually the mother) either at home (a few days before the child completed the CPT) or at the study center (the same day the child participated). Parents were asked to indicate how many hours per day (separately for a normal weekday and a weekend day) the child usually spends (a) watching movies/TV shows, (b) playing computer games, and (c) reading either autonomously (older age group) or listening to the caregivers’ reading (younger age group). Reading referred to reading for non-academic purposes (not for school). Seven response categories were presented, ranging from “never” to “more than 4 hours per day”, and parents had to choose the most appropriate one. For further analysis, the categories were transformed into durations (“never” = 0, “up to 0.5 hours per day” = 0.5, “0.5 to 1 hour per day” = 1, “1 to 2 hours per day” = 1.5, “2 to 3 hours per day” = 2.5, “3 to 4 hours per day” = 3.5, “more than 4 hours per day” = 5) and the durations on weekdays and weekend days were combined to form a weighted average (i.e., watching movies/TV shows per day = ((duration watching movies/TV shows on a weekday*5) + (duration watching movies/TV shows on a weekend day*2))/7).

Like reports on attention, parental reports on children’s media use can be inaccurate or biased. However, as with most other studies, it was unfortunately not possible in LIFE Child to implement other (more complex and expensive) methods of recording media usage times.

#### Income

The family’s household equivalent income (as an indicator of socio-economic status) was assessed in a digital questionnaire completed by a parent at home or at the study center. The questionnaire was adapted from a questionnaire used in a German nationwide survey on child health [37]. The parents were asked to report the amount of money available each month. Their responses were transformed to a score ranging from 1 (low) to 7 (high), based on categorizations defined in a representative German sample [37]. The highest score is obtained when the household equivalent income is 2800 Euros or more per person [37].

### Statistical analysis

Data were analyzed using the free software R 4.0.3 [38]. Descriptive statistics were presented in terms of means and standard deviations (for continuous variables) or total and relative frequencies (for categorical variables). T-tests or chi-squared tests were applied to assess differences in the distributions of sex and income depending on age group. Correlation analyses were performed to assess associations between the times spent with different media.

Differences in performance on the CPT depending on age, sex, and income were assessed using linear regression analyses, with the CPT outcomes (error rates, reaction time sd) included as dependent variables, and age (as continuous measure), sex (female versus male), and income (as continuous measure) included as independent variables.

Linear regression analyses were also applied to assess the associations between the durations of different types of media use, included as independent variables, and the CPT outcomes, included as dependent variables. In a first step, the independent variables were included separately. In a second step, all media use variables showing significant associations with the same CPT outcome were included in one (fully-adjusted) model. All associations were controlled for age, sex, income, and (in the older age group) subversion of the CPT (1-back slow or fast).

## Results

### Descriptive statistics

Sample characteristics are presented in Table 1. In both age groups, male participants were slightly over-represented. The distribution of sex did not differ between both age groups (*p* =.901). Similarly, the amount of children from families with the highest family income (approximately 25% in each age group) did not differ between age groups (*p* =.07). However, the average income score was significantly higher in the younger age group (M = 5.8) than in the older age group (M = 5.6, *p* <.001).

With respect to media use, watching movies/TV shows was reported most frequently (M = 1.0 h per day in the younger age group and 1.3 h per day in the older age group). Playing video games was not very popular in the younger age group (M = 0.2 h per day) but gained popularity in the older age group (M = 0.8 h per day). Listening to reading (younger age group) and reading autonomously (older age group) were performed for about 0.8 h per day, on average.

The times spent watching movies/TV shows and playing video games were significantly correlated (*r* =.34 in both age groups, *p* <.001). In the younger age group, we observed significant negative correlations between being read to and watching movies/TV shows (*r* = −.12, *p* =.010) or playing video games (*r* = −.10, *p* =.041). In the older age group, in contrast, the correlations between autonomous reading and use of electronic media were not significant (*r* = −.07 (*p* =.105) and − 0.06 (*p* =.113), respectively).

**Fig. 1:**
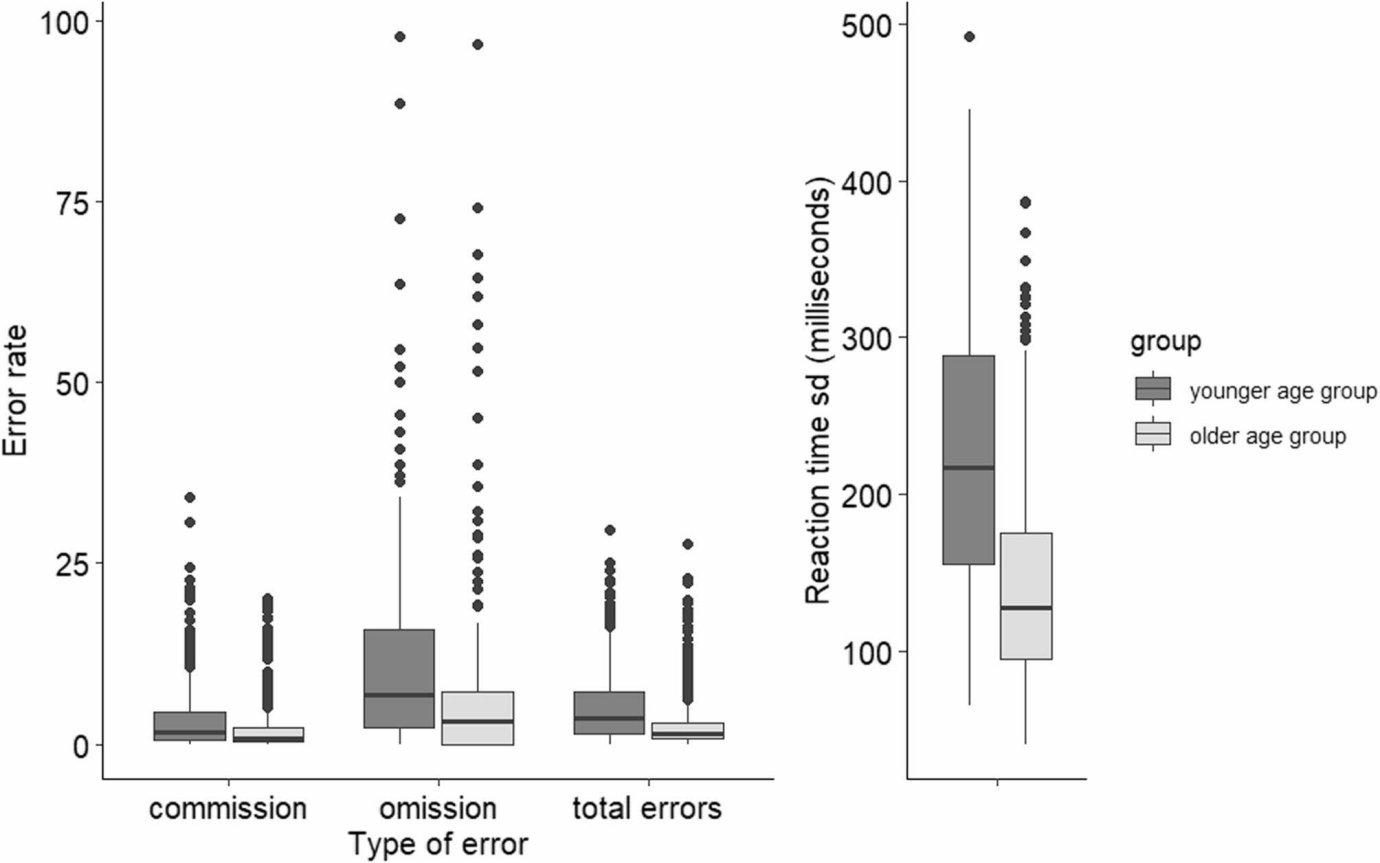
Error rates and reaction time variability in the CPT by age group. Errors of commission: Child reacts although target is not shown. Errors of omission: Child does not react although target is shown

The distributions of error rates and reaction time variability in the CPT are displayed in Fig. 1, separated by age group. Overall, error rates were rather low. Children in the younger age group made more errors than children in the older age group.

**Table 1:** Characteristics of the younger age group and the older age group

		Younger age group (*n* = 440)	Older age group (*n* = 617)
Sociodemographic characteristics			
Age	M (SD)*	5.0 (0.8)	9.1 (1.6)
Sex	n (%) male	228 (52%)	315 (51%)
	n (%) female	212 (48%)	302 (49%)
Income score	M (SD)*	5.8 (1.3)	5.6 (1.4)
Income category^a^	n (%) highest	124 (28%)	145 (24%)
Media use (hours/day)			
Watching movies/TV shows	M (SD)	1.0 (0.6)	1.3 (0.8)
Playing video games	M (SD)	0.2 (0.4)	0.8 (0.8)
Reading/listening to reading^b^	M (SD)	0.8 (0.4)	0.7 (0.6)

The younger age group completed the simple CPT, the older age group completed the 1-back CPT slow (age 6.5–9.5) or fast (age 9.5–11)

^a^highest household equivalent income: score 7/7, 2800 Euro or more (per person)

^b^In the younger age group, we assessed the time being read to; in the older age group, we assessed the time of autonomous reading

*significant difference between age groups (*p* < .05)

### Associations between CPT performance and age, sex, and income

The associations between the different CPT outcomes and sociodemographic characteristics of the children are shown in Table 2. In both the younger and the older age group, the rate of errors of all types (commission and omission) and the variation in reaction time decreased significantly with increasing age. Girls of both age groups made significantly fewer errors of commission than boys. The same tendency was observable for errors of omission, but the differences were not statistically significant. The overall error rate was significantly lower in girls in both age groups. Children from families with higher income made significantly fewer errors of commission in both age groups. In the older age group, children from families with higher income also made significantly fewer errors of omission.

**Table 2:** Associations between CPT error rates/reaction time variability (dependent variables) and age, sex, and income (independent variables) in the younger and the older age group. Boldface indicates statistical significance (*p* < .05)

	Younger age group		Older age group	
	b (95% CI)	p	b (95% CI)	p
Rate of errors of commission (false positive)				
Age	**−0.68 (−1.24**,** −0.12)**	**0.017**	**−0.30 (−0.61**,** −0.00)**	**0.049**
Sex (female)	**−1.46 (−2.37**,** −0.54)**	**0.002**	**−0.65 (−1.16**,** −0.14)**	**0.013**
income	**−0.36 (−0.72**,** −0.00)**	**0.049**	**−0.23 (−0.41**,** −0.05)**	**0.014**
Rate errors of omission (missings)				
Age	**−7.38 (−8.75**,** −6.02)**	**< 0.001**	**−2.40 (−3.32**,** −1.46)**	**< 0.001**
Sex	−1.44 (−3.68, −0.80)	0.207	−0.84 (−2.39, −0.71)	0.289
Income	−0.26 (−1.14, 0.62)	0.557	**−1.03 (−1.58**,** −0.48)**	**< 0.001**
Rate total errors				
Age	**−2.02 (−2.56**,** −1.48)**	**< 0.001**	**−0.62 (−0.95**,** −0.28)**	**< 0.001**
Sex	**−1.45 (−2.34**,** −0.57)**	**0.001**	**−0.68 (−1.24**,** −0.11)**	**0.018**
Income	−0.34 (−0.69, 0.01)	0.055	**−0.35 (−0.54**,** −0.15)**	**< 0.001**
Reaction time sd (in milliseconds)				
Age	**−52.78 (−61.18**,** −44.39)**	**< 0.001**	**−8.45 (−13.93**,** −2.98)**	**0.003**
Sex	−11.23 (−25.00, 2.54)	0.110	−3.99 (−13.13, 5.15)	0.392
Income	1.50 (−3.92, 6.91)	0.587	−2.77 (−5.99, 0.46)	0.092

The younger age group completed the simple CPT, the older age group completed the 1-back CPT slow (age 6.5–9.5) or fast (age 9.5–11)

Age, sex, and income were included simultaneously in the same model. In the older age group, associations were adjusted for test subversion (1-back slow versus fast)

### Association between CPT performance and media use/reading

**Fig. 2:**
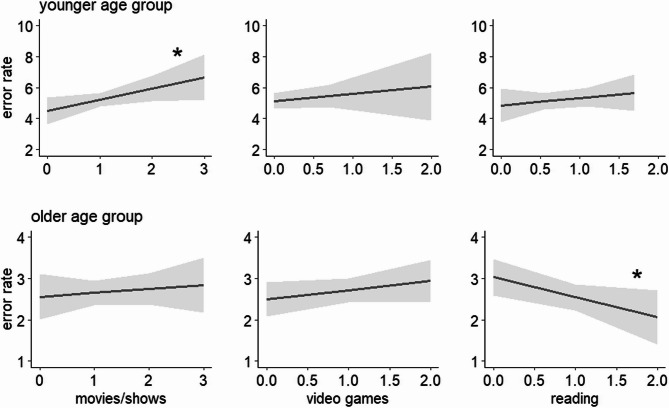
Associations (and 95% CI) between times spent for different media activities and the total error rates in the younger and the older age group. *p <.05

The associations between the CPT outcomes and the different media activities are shown in Table 3. In the younger age group completing the simple version of the CPT, watching movies/TV shows was associated with a significantly higher rate of errors of commission (reacting although the target is not shown) and a significantly higher total error rate (see Fig. 2). In contrast, being read to and playing video games showed no significant association with any type of errors.

In the older age group performing the 1-back CPT, watching movies/TV shows and playing video games were associated with a significantly higher rate of errors of omission. In a fully-adjusted model, both associations lost statistical significance (b_tv_ = 0.71 (95% CI −0.33, 1.75), *p* =.180; b_gaming_ = 0.88 (95% CI −0.19, 1.95), *p* =.108). This indicates that the effects of watching movies/TV shows and playing video games are not independent of each other. Autonomous reading was associated with a lower rate of errors of commission (which was only marginally significant) and with a significantly lower total error rate (see Fig. 2).

The reaction time variability was not significantly associated with the use of electronic media, neither in the younger nor in the older age group.

**Table 3:** Associations between CPT error rates/reaction time variability (dependent variables) and use of different media (independent variables) in the younger and the older age group. Boldface indicates statistical significance (*p* < .05)

	Younger age group		Older age group	
	b (95% CI)	p	b (95% CI)	p
Rate of errors of commission				
Movies/TV shows	**1.05 (0.32**,** 1.77)**	**0.005**	−0.13 (−0.47, 0.20)	0.435
Video games	0.05 (−1.17, 1.26)	0.938	0.05 (−0.30, 0.39)	0.786
Reading^a^	0.91 (−0.34, 2.16)	0.155	−0.43 (−0.88, 0.02)	0.062
Rate of errors of omission				
Movies/TV shows	−0.57 (−2.36, 1.22)	0.531	**1.02 (0.02**,** 2.01)**	**0.046**
Video games	2.10 (−0.88, 5.08)	0.167	**1.09 (0.08**,** 2.10)**	**0.035**
Reading^a^	−1.24 (−4.32, 1.83)	0.429	−0.89 (−2.21, 0.43)	0.178
Rate total errors				
Movies/TV shows	**0.72 (0.02**,** 1.43)**	**0.045**	−0.04 (−0.34, 0.41)	0.851
Video games	0.46 (−0.72, 1.64)	0.446	0.20 (−0.18, 0.58)	0.293
Reading^a^	0.48 (−0.74, 1.70)	0.441	** −0.50 (−0.99 **,** −0.01)**	**0.047**
Reaction time sd (in milliseconds)				
Movies/TV shows	10.04 (−0.92, 21.01)	0.072	−1.38 (−7.19, 4.43)	0.642
Video games	4.34 (−13.98, 22.66)	0.642	−1.11 (−7.02, 4.81)	0.713
Reading^a^	4.75 (−14.19, 23.68)	0.622	−6.65 (−14.35, 1.05)	0.090

All associations are adjusted for age, sex, income, and (in the older age group only) test subversion (1-back slow versus fast)

^a^In the younger age group, we assessed the time being read to; in the older age group, we assessed the time spent reading autonomously

## Discussion

This cross-sectional study explored associations between media use and performance on an attention task (CPT) in children aged 3 to 11 years. Overall, performance on the CPT was better in girls than boys, increased with increasing age, and was higher in children from families with higher income. These sociodemographic associations are in line with previous research findings regarding performance on attention tests [39] and the frequency of parent-reported symptoms of inattention/hyperactivity [40, 41].

### Screen-based media and the association with performance on the CPT

Overall, the use of electronic media in the present sample was rather high. For children at preschool or primary school, the World Health Organization (WHO) recommends to not spend more than one hour per day using screens [12]. In our sample, the average duration of watching movies/TV shows alone was already one hour per day, not only in the older age group but also in the younger age group.

Long times playing video games were significantly associated with long times watching movies/TV shows, indicating that children are often interested in different types of electronic media. In 3- to 6.5-year-old children, listening to reading was significantly associated with less frequent use of electronic media (watching movies/TV shows and playing video games). This can possibly be explained by parents’ educational concepts. In families where reading is highly valued, the use of electronic media is often less appreciated. In contrast, in the older children (6.5- to 11-year-olds), where we asked about children’s autonomous reading, reading and the use of electronic media were not related to each other.

As hypothesized, watching movies/TV shows or playing video games for long time periods was associated with more errors in the CPT. This finding is in line with results of previous studies in which attention deficits were assessed via questionnaires or parental reports [17–19] or paper-pencil-tests [21]. One explanation for this finding is that electronic media are often characterized by rapidly changing stimuli (e.g., rapidly changing images when watching movies/TV shows or playing video games). Therefore, they do not hold children’s attention for long periods of time, which makes it more difficult for them to devote themselves to a (boring) task for a long time [24]. Another explanation is that children who have difficulty concentrating for long periods of time make greater use of screen-based media, e.g., because their needs for excitement are satisfied immediately [24]. It is important to note that, given the cross-sectional design of our study, we are not able to tell which of these two possible mechanisms is more likely or whether both act in parallel.

Interestingly, the different types of electronic media use were differentially associated with different types of errors, with variations among age groups. Playing video games was associated with more errors of omission in the older age group only. Children aged 6 years and older who play video games more frequently might be used to more exciting and stimulating computer games and, therefore, not follow the rather boring CPT closely. In children younger than 6 years old, playing video games was very infrequent. This might be a reason why there was no association in that age group.

Watching movies/TV shows was associated with errors of commission in the younger age group. In the older age group, in contrast, watching movies/TV shows was associated with errors of omission, similar to playing video games. Our results indicate that not the use of one specific electronic medium (TV or gaming) but rather the use of electronic media in general may be associated with errors of omission in that age group. Comparisons with previous studies are difficult as they often focused on the use of only one media device [15–18, 20] or assessed screen time in general [19].

Overall, our findings suggest that the use of electronic media might trigger impulsive behavior in preschool-aged children and a lack of sustained attention in young school children. On the other hand, the findings might also indicate that younger children with difficulties in impulse control and older children with difficulties in sustained attention might be especially interested in using electronic media. Since the CPT tasks differed slightly between age groups (simple versus 1-back), we cannot rule out that variations in the associations are (partly) due to these differences.

Neither watching movies/TV shows nor playing video games was significantly associated with reaction time variability. This suggests that the use of electronic media and difficulties in attention stability are not inter-related in (young) children.

### Reading and performance on the CPT

On average, children of the younger age group were read to about half an hour per day. In Germany, reading aloud/shared book reading is a fixed ritual in many families, which might explain this rather long duration. The average duration of autonomous reading per day was also half an hour. The reading times were shorter than the time children spent using electronic devices. It can therefore be considered a less popular leisure activity among children.

As expected, longer durations of autonomous reading were significantly associated with overall lower error rates in the CPT. This finding is in line with a previous study showing significant associations between reading frequency and performance in an attention test [21]. The same tendencies were observed for the two error types (errors of omission versus errors of commission), but these associations were not statistically significant.

Interestingly, we observed no significant associations between listening to the caregivers’ reading and attention performance in 3- to 6.5-year-old children. This finding might suggest that only autonomous reading, but not listening to someone’s reading, is associated with improved attention performance. The type of attention might be one explanation for this: Both autonomous reading and completing the CPT require visual attention and may therefore be associated with each other. The attention required when listening to a reader, on the other hand, is more auditory. Another possible explanation is that it is not clear how attentively children actually listen to caregivers’ reading. Autonomous reading, in contrast, always requires attention.

In contrast to previous studies showing significant positive associations between reading ability and attention in children [27–31], the present study assessed the frequency of reading, not reading ability. Therefore, our results provide evidence that motivation to read (and not only reading ability) may play an important role in children’s attention. At the same time, the results may indicate that attention problems complicate the reading process and discourage children from reading. Future studies might explore whether the well-established link between reading ability and attention is mediated by the motivation to read.

As the use of electronic media, being read to or reading autonomously was not significantly associated with reaction time variability in the CPT, indicating that reading and difficulties in attention stability are not related in (young) children.

### Strengths and limitations

Strengths of the present study are the large sample size, the assessment of attention performance using a standardized computer test, the consideration of different types of media activities, and the distinctions made between children of different age groups. However, it has to be acknowledged that the associations found in this study were rather weak, indicating that the use of media is only one of many more essential factors that play a role in children’s attention. Another important limitation is the under-representation of children from families with lower income. Therefore, the results may not be generalizable to children from disadvantaged families. Another limitation is that children’s media use was reported by their parents. These reports are based on subjective perceptions and might be biased, e.g., by social desirability. In addition, not all relevant types of media use were assessed in this study. For example, the use of social media was not included. Regarding the assessment of attention, different age-adapted versions of the CPT were applied, which made direct comparisons of performance of children of different age groups difficult. Also, other versions of the CPT might have been helpful to distinguish even more aspects of attention, e.g., vigilance versus sustained attention [42]. Another limiting factor is that potentially influential characteristics such as intelligence and reading abilities were not assessed in this study and, therefore, could not be investigated as independent variables or covariates. Furthermore, it is important to mention that data were collected within the Covid-19 pandemic. Even if children were allowed to go to school, other restrictions of everyday life might have had an impact on their media use or their attention. Finally, as the data were cross-sectional, we cannot draw conclusions on causal relationships between attention and media use.

## Conclusion

The present study showed that children aged 3 to 11 years made more errors in a standardized attention task if they used electronic media more frequently. Reading, in contrast, was associated with fewer errors in children aged 6.5 to 11 years. On the one hand, these findings might indicate that reading is conducive to attentional performance, while the use of electronic media is not. On the other hand, they might also suggest that difficulties in attention can have an impact on which media activities children prefer.

## Data Availability

The datasets generated and/or analyzed during the current study are not publicly available due to ethical restrictions. The LIFE Child study is a study collecting potentially sensitive information. Publishing data sets is not covered by the informed consent provided by the study participants. Furthermore, the data protection concept of LIFE requests that all (external as well as internal) researchers interested in accessing data sign a project agreement. Researchers that are interested in accessing and analyzing data collected in the LIFE Child study may contact the data use and access committee (forschungsdaten@medizin.uni-leipzig.de).

## Abbreviations

ADHD Attention: Deficit/Hyperactivity Disorder
CPT: Continuous Performance Test
ISI: Interstimulus interval
